# Multiband multi-echo simultaneous ASL/BOLD for task-induced functional MRI

**DOI:** 10.1371/journal.pone.0190427

**Published:** 2018-02-01

**Authors:** Alexander D. Cohen, Andrew S. Nencka, Yang Wang

**Affiliations:** Department of Radiology, Medical College of Wisconsin, Milwaukee, Wisconsin, United States of America; Johns Hopkins School of Medicine, UNITED STATES

## Abstract

Typical simultaneous blood oxygenation-level dependent (BOLD) and arterial spin labeling (ASL) sequences acquire two echoes, one perfusion-sensitive and one BOLD-sensitive. However, for ASL, spatial resolution and brain coverage are limited due to the T1 decay of the labeled blood. This study applies a sequence combining a multiband acquisition with four echoes for simultaneous BOLD and pseudo-continuous ASL (pCASL) echo planar imaging (MBME ASL/BOLD) for block-design task-fMRI. A multiband acceleration of four was employed to increase brain coverage and reduce slice-timing effects on the ASL signal. Multi-echo independent component analysis (MEICA) was implemented to automatically denoise the BOLD signal by regressing non-BOLD components. This technique led to increased temporal signal-to-noise ratio (tSNR) and BOLD sensitivity. The MEICA technique was also modified to denoise the ASL signal by regressing artifact *and* BOLD signals from the first echo time-series. The MBME ASL/BOLD sequence was applied to a finger-tapping task functional MRI (fMRI) experiment. Signal characteristics and activation were evaluated using single echo BOLD, combined ME BOLD, combined ME BOLD after MEICA denoising, perfusion-weighted (PW), and perfusion-weighted after MEICA denoising time-series. The PW data was extracted using both surround subtraction and high-pass filtering followed by demodulation. In addition, the CBF/BOLD response ratio and CBF/BOLD coupling were analyzed. Results showed that the MEICA denoising procedure significantly improved the BOLD signal, leading to increased BOLD sensitivity, tSNR, and activation statistics compared to conventional single echo BOLD data. At the same time, the denoised PW data showed increased tSNR and activation statistics compared to the non-denoised PW data. CBF/BOLD coupling was also increased using the denoised ASL and BOLD data. Our preliminary data suggest that the MBME ASL/BOLD sequence can be employed to collect whole-brain task-fMRI with improved data quality for both BOLD and PW time series, thus improving the results of block-design task fMRI.

## Introduction

Functional MRI (fMRI) is a powerful, noninvasive tool to measure brain function. Two major contrasts used for fMRI are blood oxygenation-level dependent (BOLD) and arterial spin labeling (ASL). While the BOLD fMRI signal is sensitive to magnetic susceptibility fluctuations caused by changes in blood oxygenation, it is also related to changes in cerebral blood flow (CBF), cerebral blood volume (CBV), and the cerebral metabolic rate of oxygenation (CMRO2). In contrast, ASL fMRI measures blood flow changes directly by magnetically tagging blood flowing into the brain. Therefore, CBF and BOLD are commonly used to study the hemodynamic response to neuronal activity and extract information regarding the role of the microvasculature in the brain [1–5].

Sequences have been developed to obtain ASL and BOLD contrast simultaneously by collecting ASL- and BOLD-sensitive echoes in one acquisition [1–8]. These sequences have been used to assess the contributions of CBF to the BOLD response [3, 4], obtain calibrated BOLD and cerebrovascular reactivity (CVR) measurements [9, 10], and reduce total imaging time by acquiring two image contrasts in one acquisition [5]. Furthermore, simultaneous acquisition of ASL and BOLD is an important tool for neurovascular coupling measures [6] and has been applied in research of aging and stroke [7, 8]. However, ASL requires a tagging module and post-labeling delay (PLD) to allow tagged blood to flow into the brain. For pseudo-continuous ASL (pCASL), the recommended approach for ASL imaging [11], the suggested tagging time and PLD are each more than 1.5 s [11]. This approach results in long TRs and total ASL acquisition readout times that are severely limited by the short T1 relaxation of the tagged blood. In turn, it reduces the signal-to-noise ratio (SNR) and restricts the image resolution and total number of slices that can be acquired. Slices also experience varying PLD, which can affect accurate CBF quantification. Thus, most simultaneous ASL/BOLD studies acquire a 64×64 matrix, slice thicknesses >5 mm, and less than 20 slices, which can be inadequate when studying finer structures.

To address these issues, our group has recently developed a multiband (MB), multi-echo (ME) simultaneous ASL/BOLD sequence [12]. This sequence leverages two advanced techniques, MB imaging and ME imaging, to concurrently acquire whole-brain ASL and BOLD time-series with high tSNR. MB imaging, where multiple slices are excited and acquired simultaneously, can be used to increase spatial coverage and/or temporal resolution [13, 14]. MB imaging has been developed and validated for task fMRI [15] and resting-state fMRI [14, 16] and has been combined with ASL to acquire high-resolution blood flow images [17–19]. One major advantage of the addition of MB to ASL is a reduction in interslice labeling delay time differences. This is because MB imaging allows more slices to be acquired in fewer excitations compared with single band acquisitions. Fewer excitations also means T1-relaxation of the labeled blood is reduced. This leads to an increased SNR and more accurate CBF estimations [17–19]. MB-ASL has shown similar CBF estimation compared with single band ASL [19].

The MBME ASL/BOLD sequence also utilizes a multi-echo approach. In contrast to typical simultaneous ASL/BOLD sequences, which acquire only two echoes, our MBME ASL/BOLD sequence acquires four total echoes. A growing body of research indicates the image SNR, temporal SNR (tSNR), and BOLD sensitivity can be increased using ME echo-planar imaging (EPI) approaches [20–25]. The BOLD contrast is highest when the TE equals the T2* relaxation of the tissue of interest. However, T2* varies across the brain. This can be corrected for by acquiring several (>2) echoes and averaging them, weighted by the voxelwise T2*. Combining echoes in this way has resulted in improved BOLD sensitivity [20, 21, 26]. Images can be further denoised using an automated ME independent component analysis (MEICA) approach [23, 24, 27], which can robustly separate BOLD and non-BOLD components. The MEICA technique classifies independent components as either BOLD or non-BOLD depending on whether their amplitudes are linearly dependent on TE or not dependent on TE, respectively. Components deemed unrelated to BOLD fluctuations are removed from the data.

In a previous study, the MBME ASL/BOLD sequence was used to acquire resting state fMRI data in a cohort of healthy control subjects [12]. Functional connectivity strength and network size was significantly increased following MEICA denoising of the BOLD signal. Robust connectivity was also observed in well-known brain networks using ASL-derived perfusion-weighted time-series. In the current study, we applied the MBME ASL/BOLD sequence to task fMRI. Four total echoes were acquired and combined to increase the image tSNR and BOLD sensitivity and remove non-BOLD signal from the BOLD data. Furthermore, we have modified the MEICA algorithm and introduce an automated ASL denoising technique to increase the tSNR of ASL data. Task fMRI data were acquired and compared between perfusion-weighted (PW), PW denoised (PWDN), second echo (E2), multi-echo combined (MEC), and multi-echo combined, denoised (MECDN) time-series.

## Methods

### Subjects

This study was approved by the Medical College of Wisconsin Institutional Review Board, and all subjects provided written informed consent before participating. 13 healthy adult volunteers (six males, seven females; mean age = 30.2 +/- 8.5 years, ranging from 20–50 years) were recruited for this study. All subjects were right-handed. Subjects were asked to refrain from intake of caffeine before the MRI exam.

### Imaging

All imaging was performed on a General Electric 3T MR750 system with a body transmit coil and 32 channel NOVA receive head coil. A T1-weighted, magnetization-prepared rapid acquisition with gradient echo (MPRAGE) was collected and used for coregistration with the functional images. Images were acquired with the following parameters: TR/TE = 7.3/3.0ms, FA = 8°, FOV = 256mm, 1×1×1mm^3^ resolution, BW = 62.5kHz, and TI = 900ms. A T2-weighted CUBE image was also acquired with the following parameters: TR/TE = 2500/63.6ms, FA = 90°, FOV = 256mm, 1×1×1mm^3^ resolution, and BW = 125kHz.

### MBME ASL/BOLD sequence

**Fig 1:**
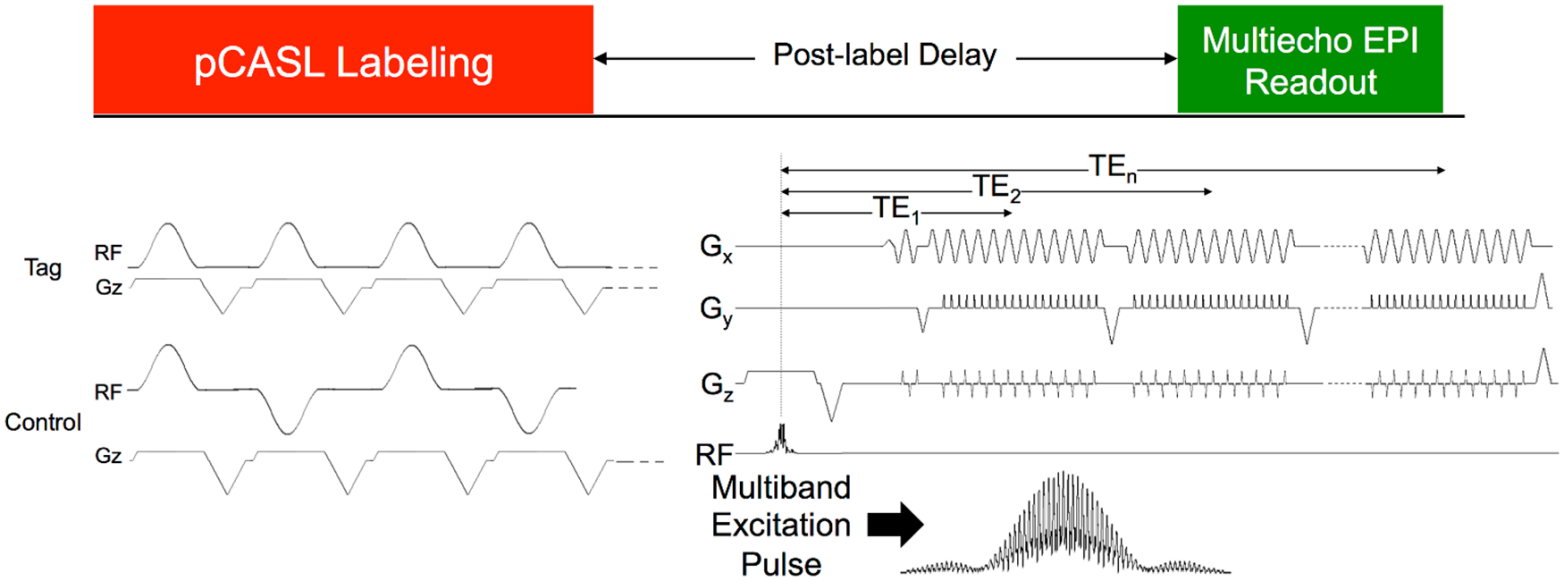
MBME ASL/BOLD pulse sequence design. The sequence consists of an unbalanced pCASL labeling train, followed by a PLD, and finally an ME EPI readout. The first echo train was acquired after the acquisition of navigator echoes through the center of k-space. The phase was then rewound to the start of k-space, and the next echo train was acquired. This was repeated three times for a total of four echoes. MB imaging was implemented by replacing the single-band excitation pulse with a MB excitation pulse. Finally, blipped-CAIPI was utilized to shift the FOV of aliased slices and reduce g-factor penalties associated with MB imaging. Reprinted from [31] under a CC BY license, original copyright 2017.

A sequence diagram for the MBME ASL/BOLD sequence is shown in Fig 1. The sequence is described in detail in [12]. In short, the sequence consists of an unbalanced pCASL tagging module [28, 29], followed by a PLD, a MB excitation, and ME gradient-echo EPI readout. Blipped-CAIPI [16] was also applied to reduce g-factor noise amplification caused by the slice-unaliasing in MB imaging, and is indicated by the z-gradient blips. In-plane acceleration was also implemented. Calibration repetitions were acquired at the start of the MBME ASL/BOLD acquisition using an interleaved approach and an MB excitation with phase-cycling [12, 30]. These repetitions were used for the subsequent unaliasing. The last repetition in each acquisition was not tagged in order to obtain an M0 image, which is the equilibrium brain tissue magnetization used to normalize the subtracted PW maps for CBF quantification.

### Functional scan protocol

Subjects underwent one task-based fMRI MBME ASL/BOLD acquisition. The sequence incorporated an unbalanced pCASL labeling scheme with labeling time = 1.5s and PLD = 1.5s. In addition, at the beginning of the MB acquisition, calibration volumes were acquired. A partial k-space acquisition was employed with a partial Fourier factor of 0.75. Additional parameters for the MBME ASL/BOLD run were as follows: number of echoes = 4; TE = 9.1,25,39.6,54.3ms; TR = 4.0 s; in-plane R = 2; MB-factor = 4; RF excitations per TR = 9 (total slices = 9×4 = 36); FOV = 240mm; resolution = 3×3×3mm^3^; matrix size = 80x80; FA = 90°; RF pulse width = 6400ms; Blipped CAIPI FOV shift = FOV/3. Scans lasted six minutes including calibration repetitions resulting in 90 functional repetitions.

During the task-based fMRI scan, subjects performed a finger-tapping task. A block-design paradigm, consisting of four alternating periods of rest and bilateral finger tapping, was applied. Rest and tapping periods lasted 10 TRs (40 sec) each. During tapping periods, subjects were instructed to tap their thumb to the other four digits sequentially at their own pace.

### Reconstruction

All image reconstruction was performed in Matlab (The MathWorks, Inc., Natick, MA, USA) and described in detail in [12]. First, Nyquist ghosting correction was performed using navigator echoes collected at the beginning of each excitation followed by echo separation. The calibration repetitions, acquired at the start of each MBME ASL/BOLD scan, were unaliased using a discrete Fourier transform and used to generate kernels for slice and in-plane unaliasing. A slice-GRAPPA algorithm [16] was implemented for slice unaliasing and applied separately for each echo. A traditional 1D-GRAPPA approach [31] was used to perform in-plane unaliasing following the slice-unaliasing procedure. Partial k-space was reconstructed using a homodyne method [32].

### Preprocessing

**Fig 2:**
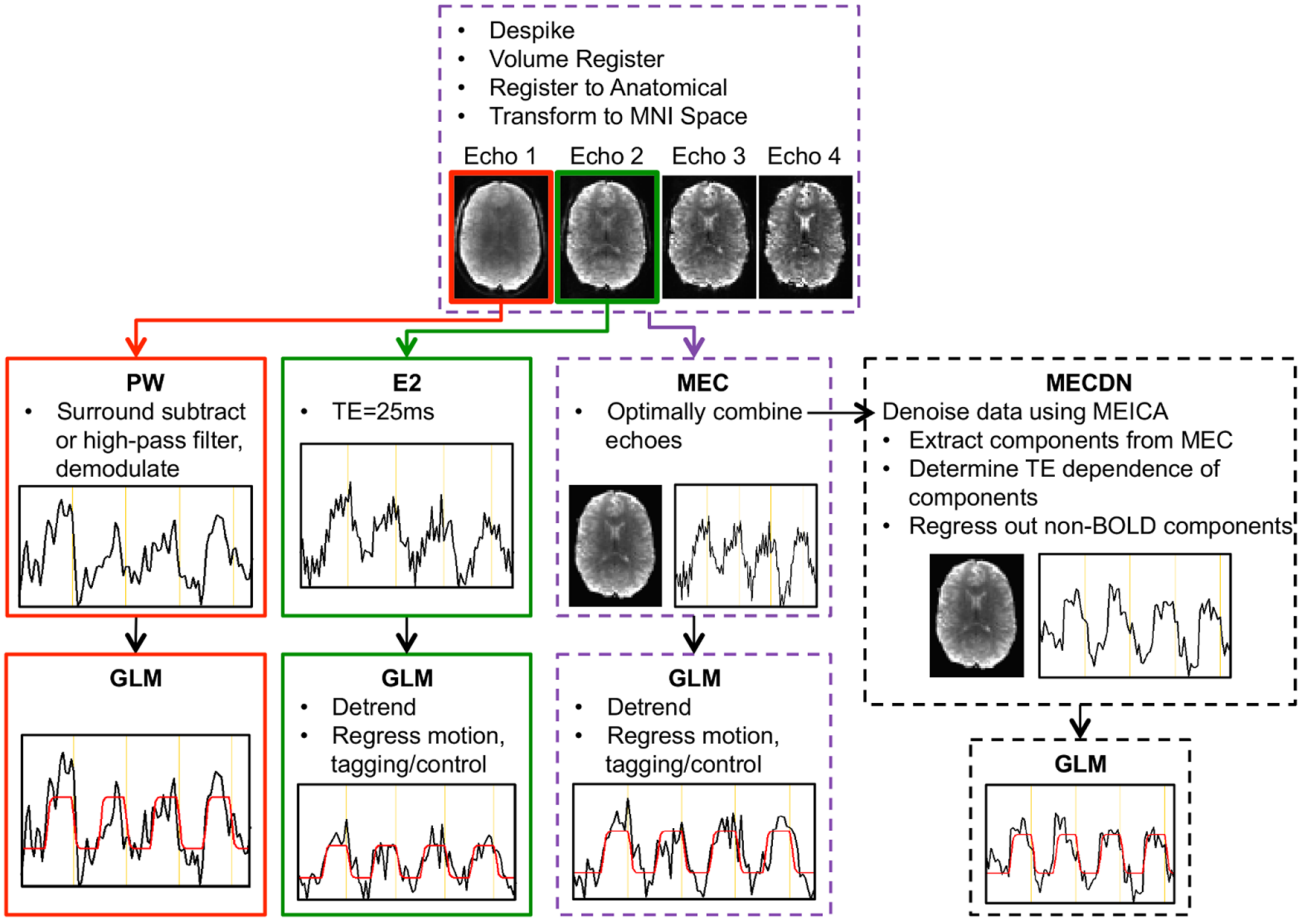
Schematic showing the processing pipeline for the ASL and BOLD echoes. The first and second echoes were processed separately to yield the PW_none_ and E2 data, respectively. Echoes were combined using a T2*-weighted approach to generate the MEC dataset. This dataset was further denoised using MEICA, resulting in the MECDN dataset. No additional regression was performed in the GLM for the PW and MECDN datasets. Example activation curves and model fits are shown for the different datasets.

The fMRI data processing pipeline is shown in Fig 2. Preprocessing was performed on each echo separately using AFNI [33, 34] (https://afni.nimh.nih.gov/afni) and FSL (http://fsl.fmrib.ox.ac.uk/fsl/fslwiki) [35]. Anatomical segmentation and registration was performed in SPM. First, the MPRAGE image was segmented into gray matter (GM), white matter (WM), and cerebrospinal fluid (CSF). The gray matter and white matter segmentations were then combined resulting in a brain-only MPRAGE image. This image was then transformed to Montreal Neurological Institute (MNI) space using a nonlinear registration algorithm. Motion was estimated for the first echo and those estimates were applied to the subsequent echoes using *3dvolreg* in AFNI. Echoes were then coregistered to the anatomical MPRAGE image using *epi_reg* in FSL [36] with an affine registration with 12 degrees of freedom. Next the functional data was transformed to MNI space via *applywarp* in FSL using the transformation matrix output from the MPRAGE-MNI registration and normalization.

### Multi-echo combination and denoising

All four echoes were combined using the T2*-weighted approach [26, 37]. First, the voxelwise mean across time of each individual echo dataset was used to estimate the signal immediately after excitation, $\bar{S_{0}}$, and the voxelwise $T_{2}^{*}$, $\bar{T_{2\left(fit\right)}^{*}}$ using log linear regression (Eq 1). The voxelwise $\bar{T_{2\left(fit\right)}^{*}}$ was then used to determine the weights, $w(T_{2}^{*})$ (Eq 2), which were used in a weighted summation of the echoes. *TE*_*n*_ represents the n^th^ echo time.

$$S\left(TE_{n}\right)\;=\;\bar{S_{0}}\cdot exp\left(-\left(1/\bar{T_{2(fit)}^{*}}\right)\cdot TE_{n}\right)$$ (1)

$$w(T_{2}^{*})\;=\;\frac{{TE}_{n}∙exp(-{TE}_{n}/\bar{T_{2\left(fit\right)}^{*}})}{\sum_{n}{TE}_{n}∙exp\;(-{TE}_{n}/\bar{T_{2\left(fit\right)}^{*}})}$$ (2)

Following the echo combination, the data were denoised using the automated MEICA technique in afni and the *meica*.*py* plugin (*v2*.*5)*. Default parameters were used except for the “daw” parameter, a weight use to control ICA dimensionality, which was raised from 10 to 20 to account for the low number of timepoints. This method is described in detail elsewhere [23, 24, 27] and classifies independent components as BOLD or non-BOLD based on whether their amplitudes are linearly dependent on TE [23, 24, 27]. Components deemed non-BOLD were removed from the data. For all cases, the tag-control modulation was identified as a non-BOLD component and removed from the data.

### ASL processing

BOLD signal is known to contaminate the PW signal [38–40]. Liu et al showed there are two components of this BOLD contamination [38]. The first component is a multiplicative term related to the non-zero echo time of the sequence. The second component is a spurious component that can be reduced by a low pass filter. These filtering techniques however, are not perfect [38, 40].

#### Perfusion-weighted denoising

**Fig 3:**
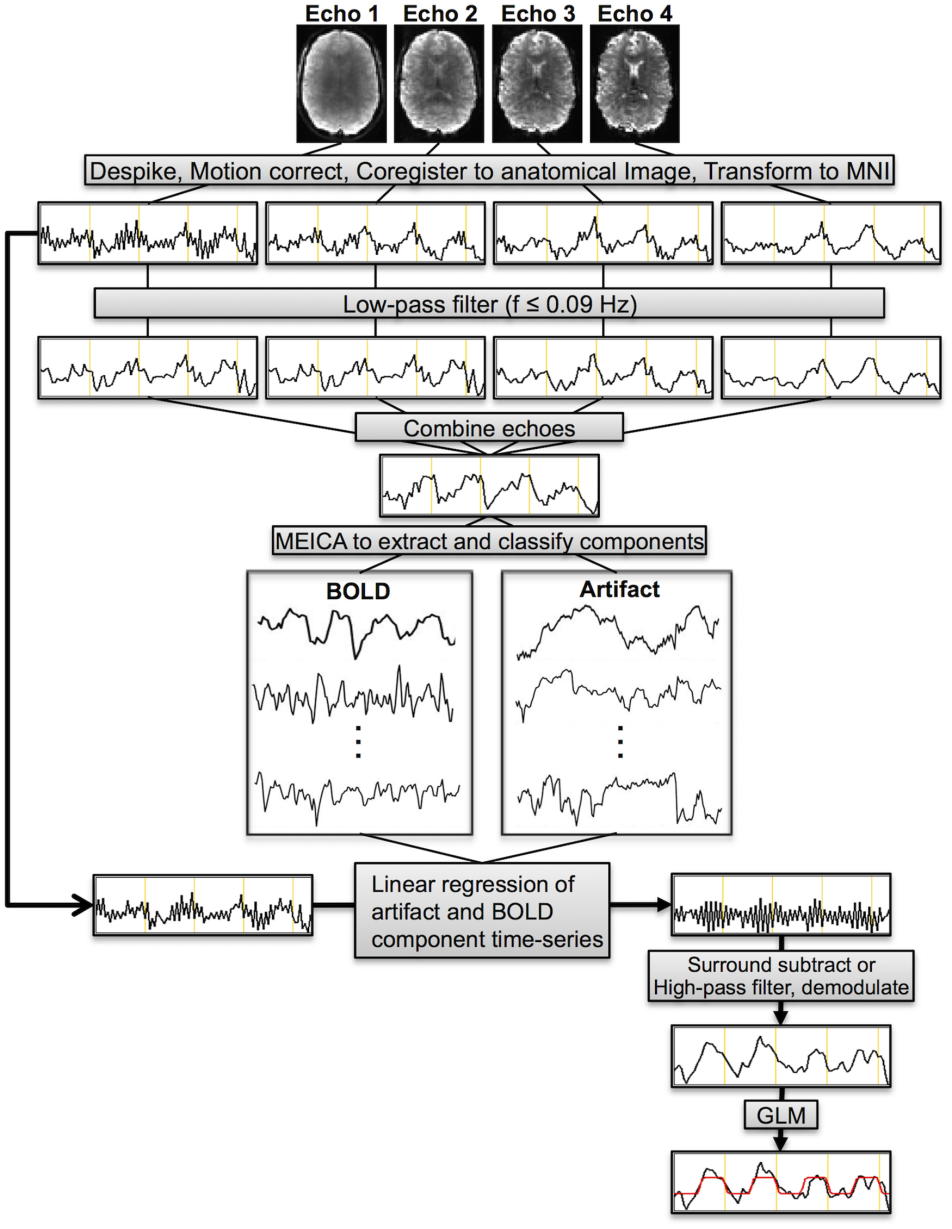
Schematic showing the perfusion-weighted denoising procedure. Echoes were first despiked, volume registered, and coregistered to MNI space. Each echo was then individually low-pass filtered at f ≤ 0.09 Hz. Echoes were then combined using a T2*-weighted approach. This low-pass filtered, multi-echo combined dataset was fed into the MEICA algorithm, which extracted independent components and classified them as artifact, BOLD, or indeterminate. The BOLD and artifact components were regressed from the unfiltered first-echo data resulting in a denoised first-echo dataset. Surround subtraction and high-pass filtering followed by demodulation were performed on this data leading to denoised PW datasets. A GLM was employed on this data to determine activation.

Therefore, we have further modified the MEICA algorithm in order to denoise the first-echo images by removing both the artifact *and* BOLD components from the signal prior to generating the PW time-series (i.e. surround subtraction and high-pass filtering and demodulation). First, data from all echoes was low-pass filtered using a band-pass filter (*3dTproject* in afni) with a maximum frequency of 0.09 Hz. This frequency is below the label-control oscillation frequency (for TR = 4s, f = 1/(2*TR) = 0.125 Hz), the main perfusion frequency (0.1125 Hz), and the second harmonic of the perfusion signal (0.1 Hz). The perfusion frequencies were determined by subtracting the task frequency (1/80s = 0.0125 Hz) from the label-control oscillation frequency. Filtering had the effect of removing PW signal oscillations from the data. Without low-pass filtering, one or more ASL components, characterized by these label/control oscillations, were identified. These components tended to be classified as artifacts and would be removed from the data, significantly reducing the tSNR of the subsequently calculated PW time-series. Next, the original MEICA algorithm was run, as described above, using the low-pass filtered echoes. This procedure combined the echoes using the T2* weighted approach [26, 37], extracted independent components, and classified those components as BOLD, artifact, and indeterminate. The BOLD and artifact components were then regressed out of the original, unfiltered first-echo data. A flow chart showing the PW denoising algorithm is shown in Fig 3.

The PW denoising procedure relies heavily on the original MEICA algorithm; however, there are two key differences between the techniques. First, a temporal filtering procedure is applied prior to MEICA to remove perfusion-related signal oscillations. The original MEICA algorithm is then run without modification on this data. Second, whereas the original MEICA algorithm removes non-BOLD components from the combined ME time-series and keeps BOLD components, here we remove artifact *and* BOLD signals from the unfiltered, heavily perfusion weighted first-echo.

An additional denoising procedure was employed where only the artifact components were removed from the first-echo time-series. This was compared to the data where both artifact and BOLD components were regressed to determine whether regressing the BOLD signal provided an added benefit over regressing only the artifact components. Furthermore, to verify PW signal was not being removed by the denoising procedure, the mean and temporal standard deviation of the PW signal in gray matter were analyzed.

#### Perfusion-weighted time-series

A PW time-series was generated for both the non-denoised and denoised first-echo data using two common, related, approaches: by surround subtracting label and control images (SS) [41] and by high-pass filtering the data followed by demodulation (HD) [4, 42]. Both these approaches are equivalent to demodulation followed by low-pass filtering, but differ in the filtering frequency. HD data was high-pass filtered with a minimum frequency of 0.09 Hz for the reasons described above. The modulated ASL component is present only above this frequency. Thus, high-pass filtering above this frequency separates the modulated ASL signal from the BOLD component present below 0.09Hz [4].

### fMRI processing

The above procedures resulted in three BOLD and six PW datasets for each scan that underwent further processing for fMRI analyses: E2 (second echo, TE = 25ms), combined multi-echo (MEC), MEC after MEICA denoising (MECDN), PW without denoising for the SS and HD data (PW_ss,none_ and PW_hd,none_), PW denoised with artifact components regressed for the SS and HD data (PWDN_ss,art_ and PWDN_hd,art_), and PW denoised with artifact and BOLD components regressed for the SS and HD data (PWDN_ss,art+BOLD_ and PWDN_hd,art+BOLD_). All data were blurred with a 4.5 mm full width at half maximum (FWHM) Gaussian kernel. For the E2 and MEC data, the six rigid-body motion parameters derived from the motion correction processing were regressed out of the data, and label-control oscillations were regressed out of the data by including a column of alternating −1s and 1s in the design matrix.

### Statistical analysis

A general linear model (GLM) was used for the task-based fMRI analysis using *3dDeconvolve* in afni. For the E2 and MEC data, in addition to the nuisance regression of motion parameters and the label-control sequence, the model included detrending with a third-degree polynomial. This was not necessary for the MECDN data where MEICA removed the low frequency drifts and motion-related artifacts. Individual activation maps were thresholded at an uncorrected p<0.01. For the MEICA denoised BOLD and ASL data, the number of degrees of freedom was reduced by the number of components regressed from the data on an individual subject basis. For the MECDN data, an average of 43.1 +/- 7.7 total components were identified, and the number of regressed components ranged from 10 to 24, with a mean of 14.9 +/- 4.6. For the PW data, an average of 32.9 +/- 5.6 total components were found. For the PW data with artifact components removed, the number of regressed components ranged from ranged from 4 to 18, with a mean of 11.2 +/- 3.9. Finally, for the PW data with artifact and BOLD components removed, the number of regressed components ranged from 13 to 33, with a mean of 22.8 +/- 5.3. For the BOLD data, following *3dDeconvolve*, a restricted maximum likelihood (REML) model (*3dREMLfit)* was used to model temporal autocorrelations in the data. This program uses an ARMA(1,1) to model the time-series noise correlation in each voxel. ASL data have been shown to not have significant temporal autocorrelation, so this model was not used for the PW data [40].

Group maps were calculated for each dataset (E2, MEC, MECDN, PW_ss,none_, PW_hd,none_, PWDN_ss,art_, PWDN_hd,art_, PWDN_ss,art+BOLD_, and PWDN_hd,art+BOLD_) using a one-sample t-test. Group maps were thresholded at p<0.005 with cluster-size thresholding to correct for multiple comparisons. The minimum cluster size was determined using the recommended approach from Cox et al to reduce the family wise error rate [43]. First, spherical autocorrelation parameters (acf) were estimated using the residuals from the t-test and 3dFWHMx in afni. These parameters were then averaged for the BOLD and ASL data separately to create one set of acf parameters for the BOLD and ASL data. Parameters were then fed into afni’s *3dClustSim* program to determine minimum cluster sizes for α<0.05. The minimum cluster size for the BOLD data was 182 and for the ASL data was 131.

For each subject and dataset, the tSNR was computed on a voxelwise basis and defined as the mean signal divided by the standard deviation of the noise across the time-series. For the BOLD data, the mean tSNR was extracted from a whole-brain mask. For the PW data, the mean tSNR was extracted from the GM segmentation. In addition, the mean and maximum t-scores of the fMRI task were computed in activated voxels using an overlap mask that was created for each subject from voxels active in all BOLD and all PW datasets separately. Noise signal was defined as the residual between each voxel’s best fit to the model and the signal itself. Statistical comparisons between mean values were made using a Bonferroni-corrected paired t-test to compare across datasets with significance set at p < 0.05.

### CBF/BOLD relationship

For most simultaneous ASL/BOLD studies, the ASL signal is used to provide a better interpretation of the activation, instead of another way to compute activation. To that end, the relationship between the ASL and BOLD activation responses was evaluated. Mean signal was extracted for E2, MECDN, PW_ss,none,_ PW_hd,none_, PWDN_ss,art+BOLD_, and PWDN_hd,art+BOLD_ data from an overlap mask consisting of voxel active for all six datasets (uncorrected p < 0.01). The time series were converted to percent signal change by dividing by the mean of the residual time-series from the GLM. The four activation blocks for each dataset were then averaged. The activation magnitude was computed as the mean of the middle five time points from the activation. The ratio between CBF and BOLD activation magnitude was computed for the E2 and MECDN data vs. the PW and PWDN datasets respectively. The relationship between these ratios and baseline CBF was also computed.

Finally, CBF/BOLD coupling was computed for the same datasets. CBF/BOLD coupling was assessed by correlating the signals from the E2 and MECDN datasets to the PW and PWDN data respectively on a voxelwise basis using Pearson correlation. The correlation maps were thresholded at an uncorrected p<0.01 and the mean correlation values were extracted. The correlation maps were then averaged across subjects to create group CBF/BOLD coupling maps.

## Results

### Data quality

**Fig 4:**
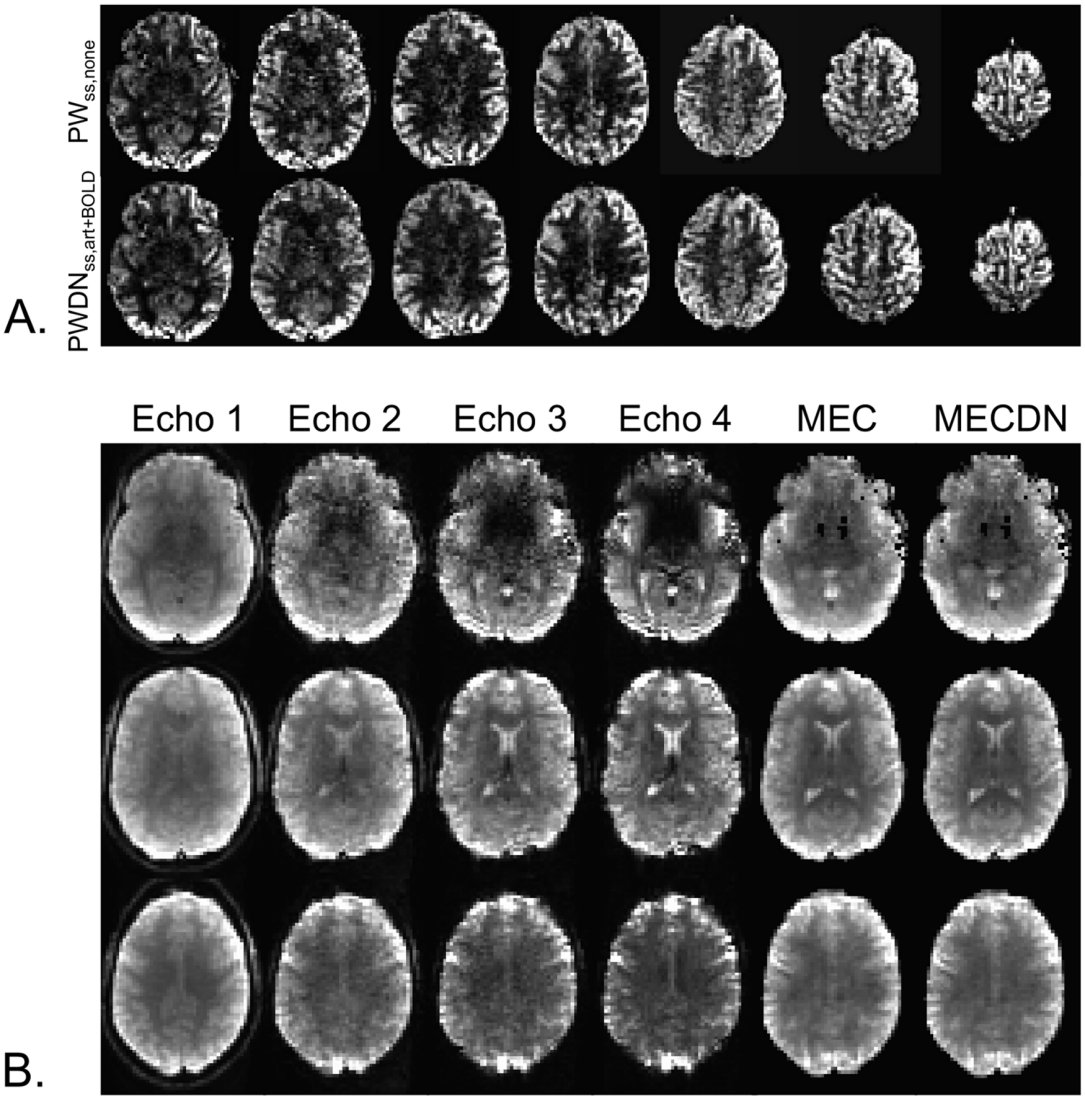
Representative PW and BOLD datasets. (A) Mean PW_ss,none_ (top) and PWDN_ss,art+BOLD_ data (bottom) images. These images were created by averaging and subtracting the label images from the control images. MB imaging allows for the collection of whole-brain images in a relatively short readout time reducing T1 effects. (B) Example individual echo, MEC, and MECDN images from a single time point from one subject. Image quality improves with echo combination.

**Fig 5:**
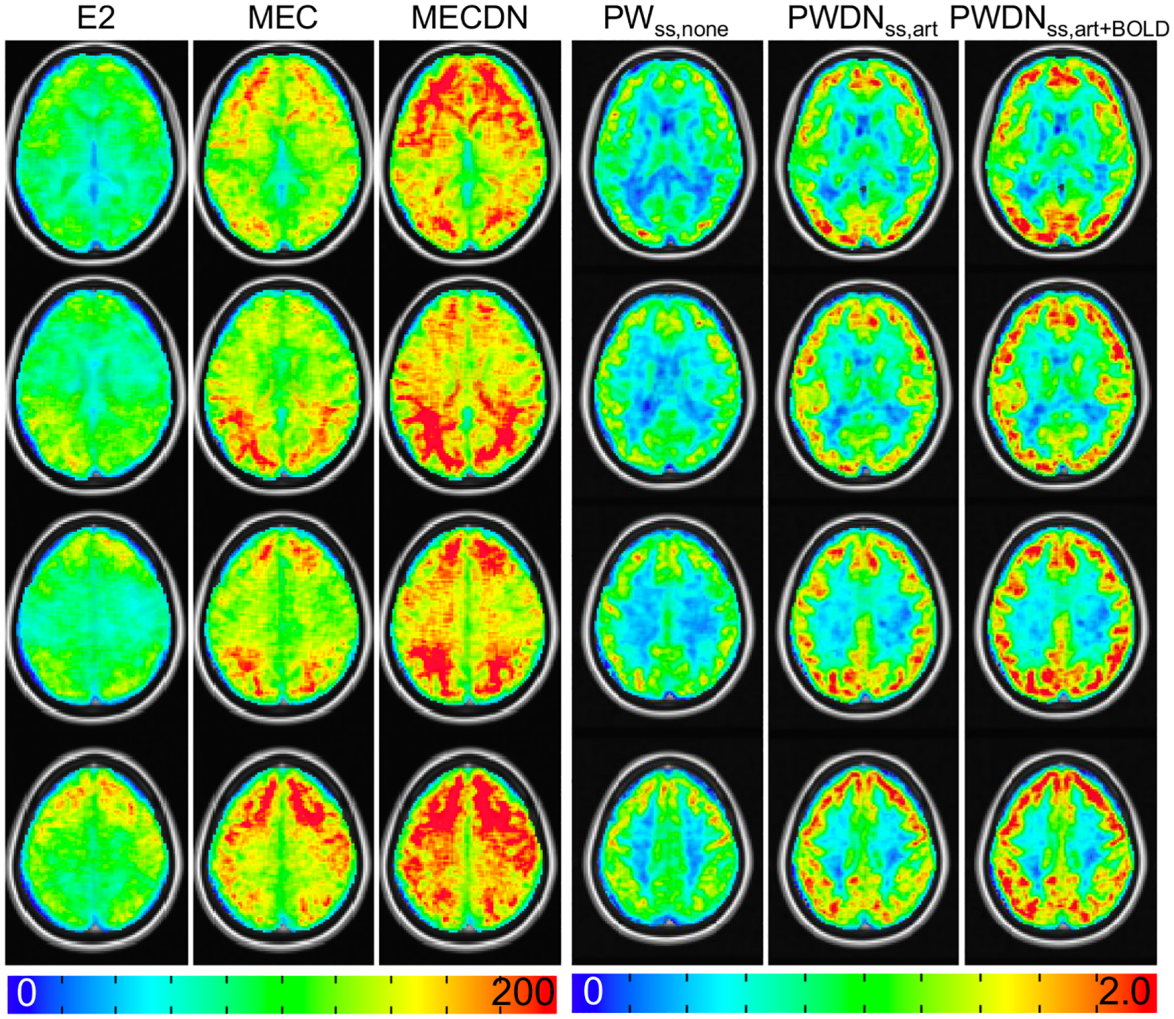
Group tSNR maps. The tSNR significantly increased from the E2 to MECDN data (p<0.001). For the PW data, tSNR maps are shown for the SS data. The tSNR for the PWDN_ss,art+BOLD_ significantly increased compared to the PWDN_ss,art_ and PW_ss,none_ data (p<0.001).

Representative individual echo, MEC, MECDN, and mean PW_ss,none_ and PW_ss,art+BOLD_ images are shown in Fig 4. Image quality was better for the MEC and MECDN data compared to any individual echo. The PW_ss,art+BOLD_ data was less noisy compared to the PW_ss,none_ data. This is most noticeable in the white matter. Group-averaged tSNR is shown in Fig 5. Quantitative results are shown in Table 1 and mirrored the results from the previously published resting state MBME ASL/BOLD study [12, 30]. Specifically, whole-brain tSNR significantly increased for the MEC data compared to the E2 data (p<0.001) and for the MECDN data compared to the MEC and E2 data (p<0.001). In addition, tSNR was significantly increased for the PWDN_ss,art+BOLD_ data compared to the PWDN_ss,art_ and PW_ss,none_ data (p<0.001) and for the PWDN_ss,art_ vs. PW_ss,none_ data (p<0.001). The same trend was observed for the HD data with PWDN_hd,art+BOLD_ tSNR increased compared to the PWDN_hd,art_ and PW_hd,none_ data (p<0.001) and for the PWDN_hd,art_ vs. PW_hd,none_ data (p<0.001).

**Table 1:** Group averages for quantitative metrics.

	Whole-brain tSNR	Mean t-score Overlapping Voxels	Max t-score Overlapping Voxels
E2	46.7 (6.2)	4.16 (0.39)	10.40 (3.41)
MEC	78.8 (9.5)	4.61 (0.63)	10.26 (3.34)
MECDN	104.4 (9.6)	5.38 (0.88)	10.65 (3.01)
Statistics	ME>SE***	ME>SE**	
	MEDN>SE***	MEDN>SE**	N.S.
	MEDN>ME***	MEDN>ME*	
PW_ss,none_	1.49 (0.29)	3.68 (0.17)	9.72 (2.18)
PWDN_ss,art_	1.71 (0.33)	3.95 (0.31)	10.48 (2.47)
PWDN_ss,art+BOLD_	1.88 (0.44)	4.30 (0.27)	11.79 (2.20)
Statistics	PWDN_ss,art+BOLD_ > PW_ss,none_***	PWDN_ss,art+BOLD_ > PW_ss,none_***	PWDN_ss,art+BOLD_ > PW_ss,none_***
	PWDN_ss,art+BOLD_ > PWDN_ss,art_***	PWDN_ss,art+BOLD_ > PWDN_ss,art_***	PWDN_ss,art+BOLD_ > PWDN_ss,art_**
	PWDN_ss,art_ > PW_ss,none_***	PWDN_ss,art_ > PW_ss,none_**	PWDN_ss,art_ > PW_ss,none_*
PW_hd,none_	1.69 (0.28)	4.88 (0.31)	11.66 (1.61)
PWDN_hd,art_	1.77 (0.32)	5.00 (0.33)	12.01 (1.50)
PWDN_hd,art+BOLD_	1.80 (0.31)	5.04 (0.31)	12.23 (1.37)
Statistics	PWDN_hd,art+BOLD_ > PW_hd,none_***	PWDN_hd,art+BOLD_ > PW_hd,none_***	PWDN_hd,art+BOLD_ > PW_hd,none_*
	PWDN_hd,art+BOLD_ > PWDN_hd,art_***	PWDN_hd,art_ > PW_hd,none_**	PWDN_hd,art_+BOLD > PWDN_hd,art_*
	PWDN_hd,art_ > PW_hd,none_***		

Note: Data is presented as mean (standard deviation). Mean and max t-scores were extracted from active voxels that overlapped for the BOLD data (E2, MEC, and MECDN) and PW data (PW_ss,none_, PWDN_ss,art_, PWDN_ss,art+BOLD_, PW_hd,none_, PWDN_hd,art_, and PWDN_hd,art+BOLD_) separately. Individual statistical maps were thresholded at an uncorrected p<0.01. Abbreviations: tSNR = temporal signal to noise ratio; E2 = single echo (second echo, TE = 25ms); MEC = multi-echo combined; MECDN = multi-echo combined and denoised; PW = perfusion-weighted; PWDN = perfusion-weighted denoised; SS = surround subtracted; HD = high-pass filtered, demodulated.

*** = p<0.001,

** = p<0.01,

* = p<0.05, Bonferroni-corrected.

No significant differences in mean PW signal were seen between PW_ss,none_, PWDN_ss,art_, or PWDN_ss,art+BOLD_ datasets (26.4±4.5, 26.5±4.5, 26.4±4.5 respectively) or PW_hd,none_, PWDN_hd,art_, or PWDN_hd,art+BOLD_ datasets (12.6±2.2, 12.6±2.2, 12.7±2.2 respectively). The increase in tSNR was driven by a significant decrease in the standard deviation after denoising for both the SS and HD datasets.

### Task fMRI

**Fig 6:**
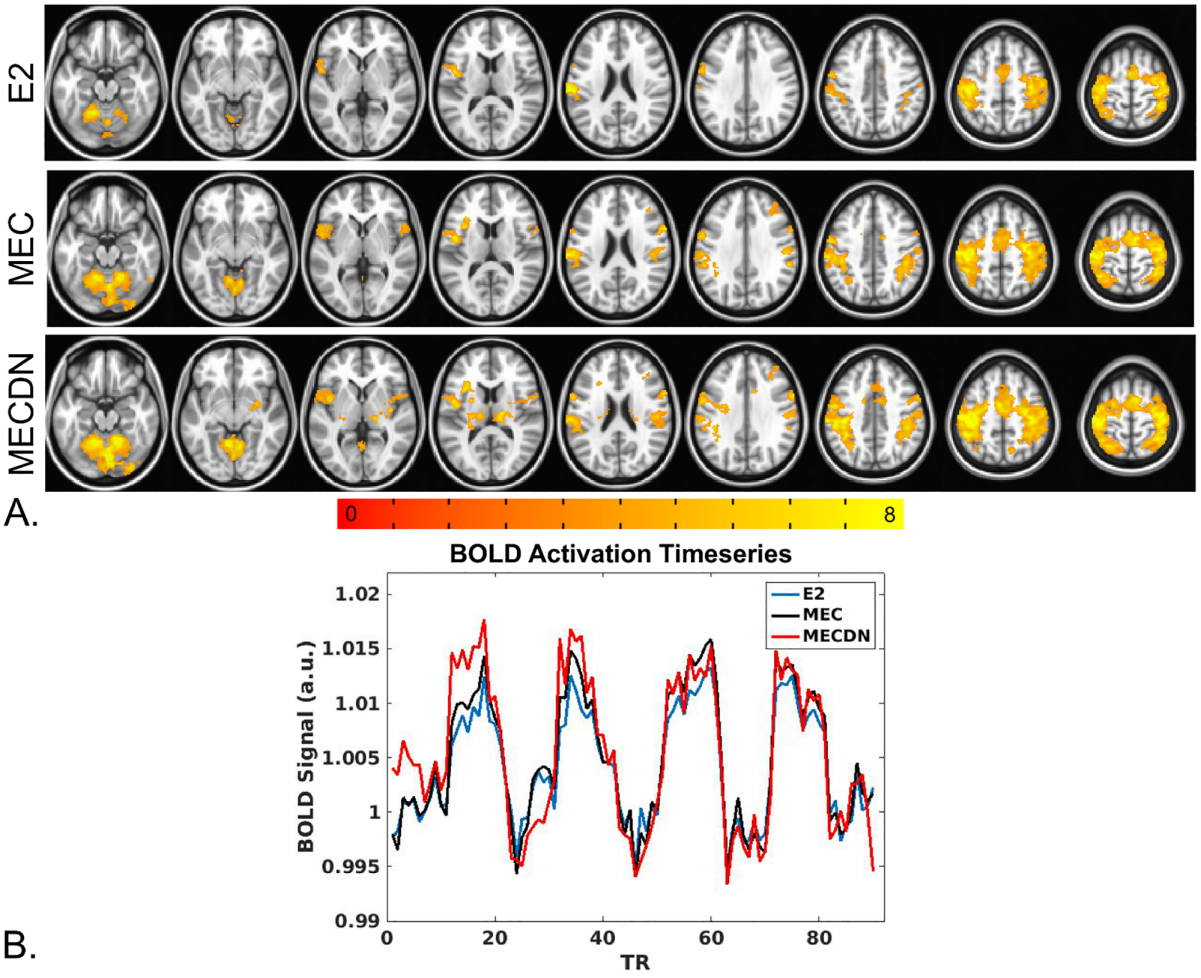
Finger-tapping task BOLD results. (A) Robust bilateral activation was seen in the motor cortex, including the pre and postcentral gyrus, medial frontal gyrus, and cerebellum for all datasets. Increased activation was observed for the MEC data compared to E2 data and for the MECDN data compared to the MEC and E2 data. Activation was also observed in subcortical areas for the MECDN data. All maps were thresholded at p<0.005 and cluster corrected with a minimum cluster size of 182 voxels (α<0.05). (B) Average BOLD time-series extracted from a mask of voxels active for all BOLD datasets.

BOLD results from the group finger tapping fMRI analysis are shown in Fig 6. Fig 6A shows the results of the group analysis across subjects for the E2, MEC, and MECDN. The MECDN dataset detected the most activation, followed by the MEC and E2 datasets. Fig 6B shows mean signal curves from one representative subject in active voxels for the E2, MEC, and MECDN datasets. An ANOVA comparing datasets did not show significant differences.

**Fig 7:**
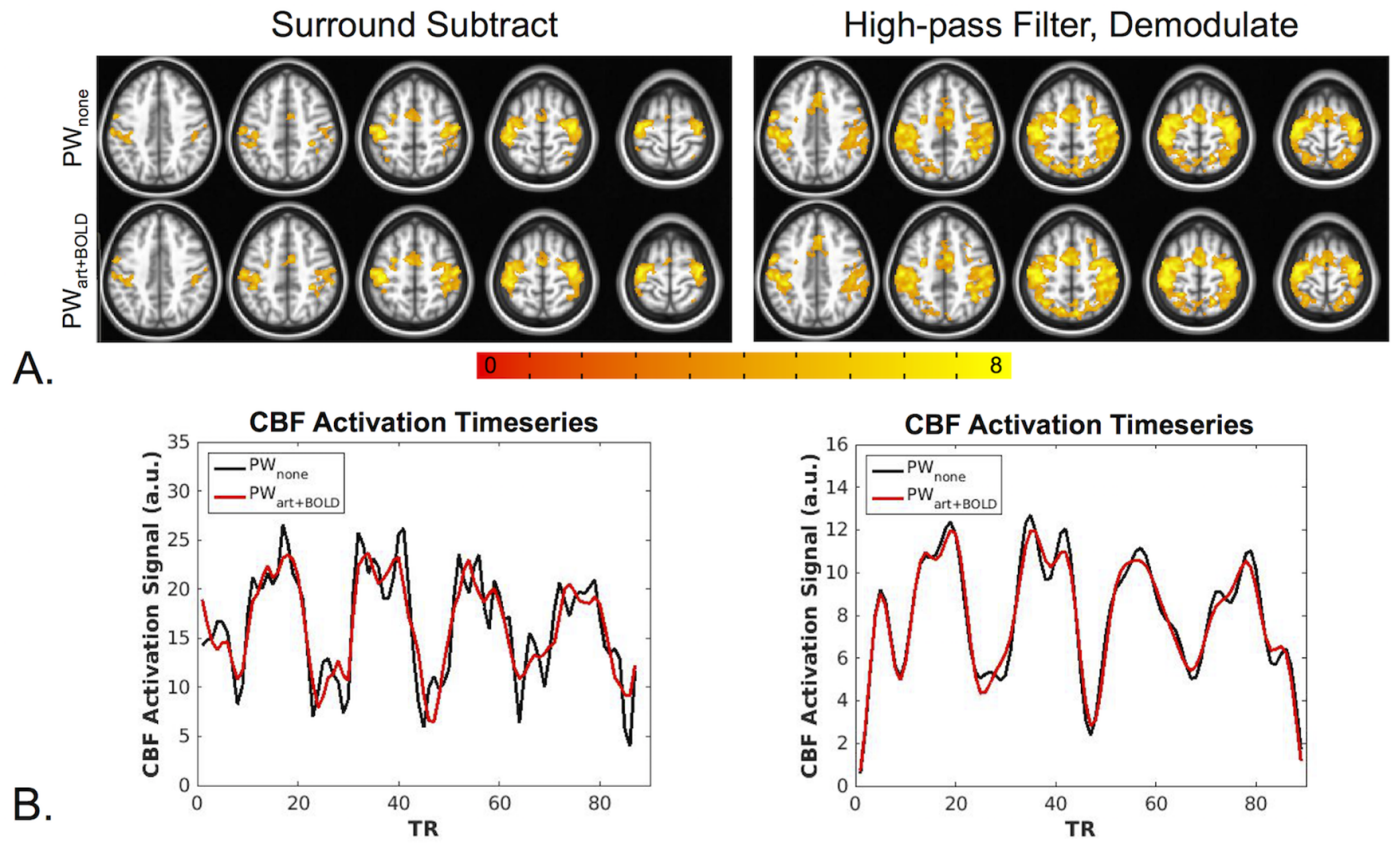
Finger-tapping task PW results. (A) For the SS results (left), bilateral activation was observed in the motor cortex for the PW_ss,none_ and PWDN_ss,art+BOLD_ data. An increased activation area was seen for the PWDN_ss,art+BOLD_ data compared to the PW_ss,none_ data. The HD data (right) showed increased activation compared to the SS data, however no differences were seen between the denoised and non-denoised data the. All maps were thresholded at p<0.005 and cluster corrected with a minimum cluster size of 131 voxels (α<0.05). (B) Average SS PW signal from one representative subject (left) and average HD PW signal from the same subject (right). All PW signal was extracted from a mask of voxels active for all PW datasets. The denoised SS time-series appear less noisy with less variance compared to the non-denoised time-series. This effect is less apparent for the HD data.

PW results are shown in Fig 7. Additional activation was observed for the PWDN_ss,art+BOLD_ data compared to the and PW_ss,none_ data (Fig 7A, left); however, no appreciable differences were observed for the PWDN_hd,art+BOLD_ data compared to the PW_hd,none_ data (Fig 7A, right). In addition, increased activation was seen for the HD data compared to the SS data. Fig 7B shows mean activation time-series from a representative subject for SS (left) and HD data (right). The SS denoised time-series appears markedly cleaner with less variance than the non-denoised SS time-series. A slight improvement can be seen for the denoised HD time series compared to the non-denoised time series.

Quantitative results are shown in Table 1. In summary, the mean t-score in active voxels was significantly increased for the MECDN and MEC data compared to the E2 data and for the MEC data compared to the E2 data. Mean t-score was also increased for the PWDN_ss,art+BOLD_ data compared to the PWDN_ss,art_ and PW_ss,none_ data and for the PWDN_hd,art+BOLD_ data compared to the PW_hd,none_ data. Maximum t-score in active voxels was not significantly different for the BOLD data, but increased with denoising for the PW data.

### CBF/BOLD relationship

**Fig 8:**
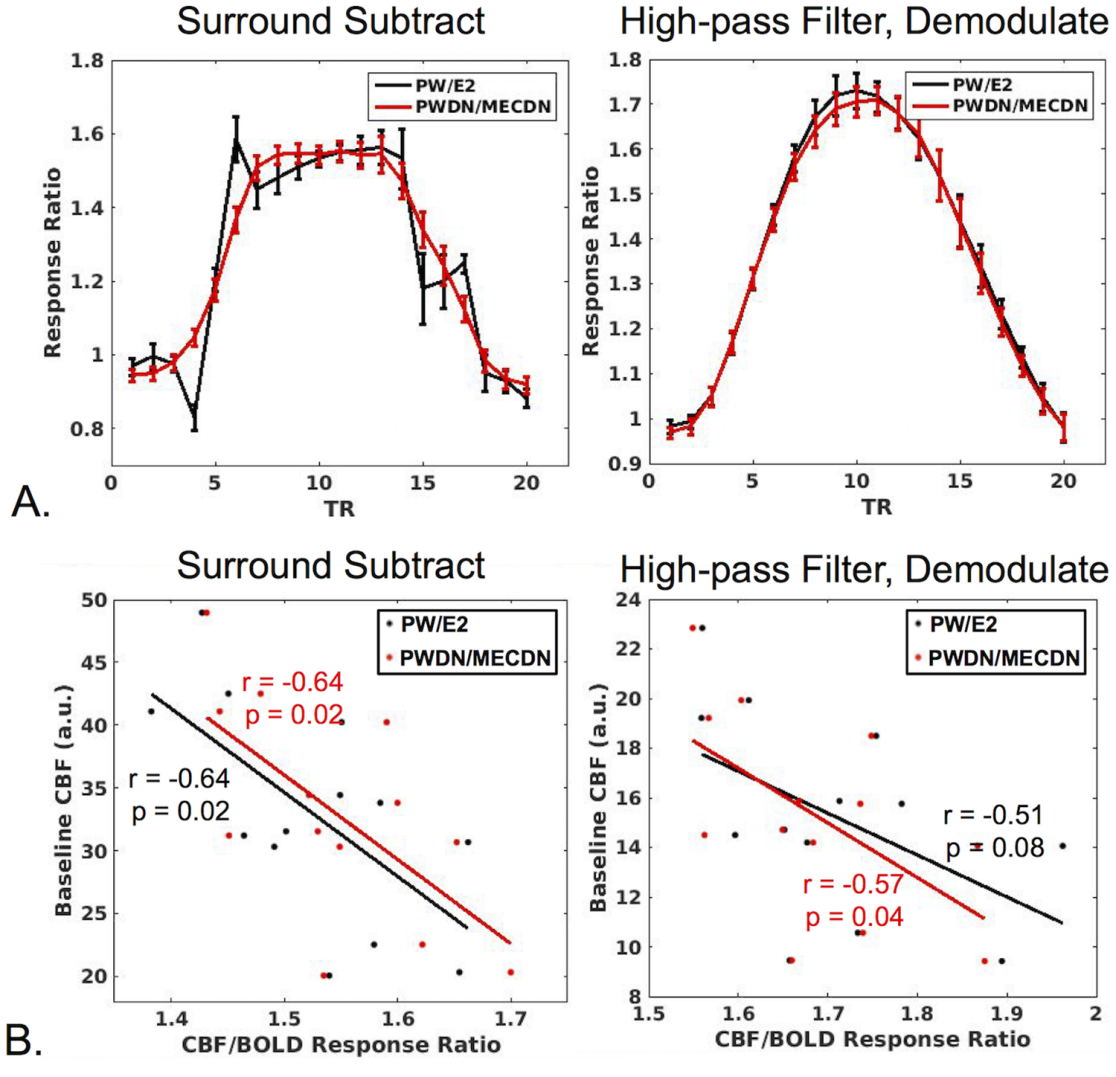
CBF/BOLD relationship. (A) The mean ratio of CBF to BOLD signal is plotted across subjects in active voxels (CBF/BOLD response ratio) for SS (left) and HD data (right). The response ratio was examined for non-denoised (PW/E2) and denoised data (PWDN/MECDN). No significant difference was observed between the non-denoised and denoised response ratios averaged across the middle five activation TRs (TR #s 8–12). (B) CBF/BOLD response ratio plotted against baseline CBF for SS (left) and HD data (right). A significant negative correlation was observed between the CBF/BOLD ratio and mean baseline CBF for all processing schemes except for HD, PW/E2 which trended toward significance.

**Fig 9:**
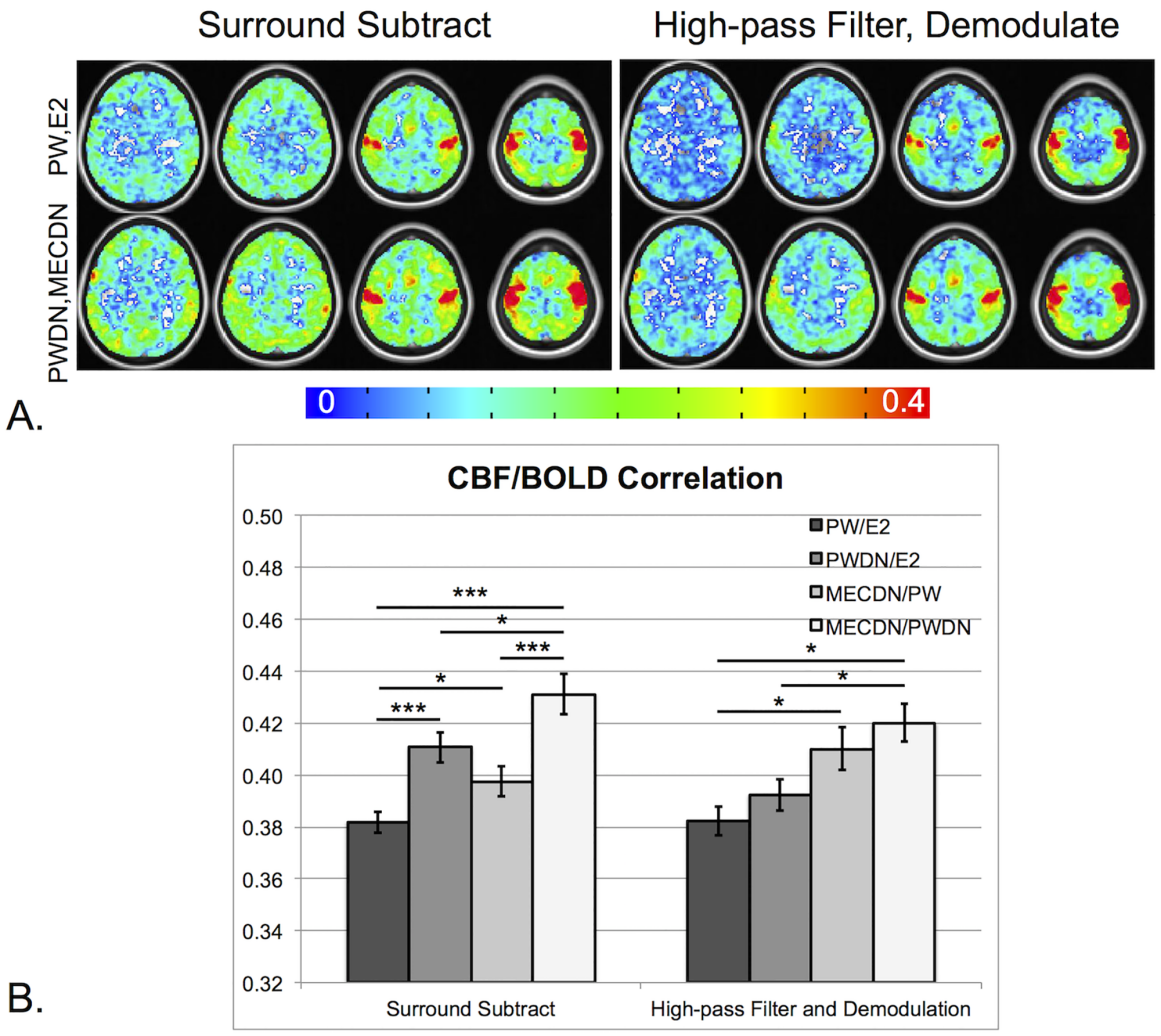
BOLD/CBF coupling. (A) Group averaged correlation between CBF and BOLD time-series for non-denoised (PW,E2) and denoised (PWDN,MECDN) data for SS (left) and HD data (right). In general, CBF-BOLD correlation increased with denoising. This was confirmed quantitatively (B) where the mean correlation extracted from a mask of significantly correlated voxels was highest for fully denoised datasets (PWDN,MECDN). *** = p<0.001, ** = p<0.01, * = p<0.05, Bonferroni-corrected.

No differences were seen between processing schemes for the CBF/BOLD response ratio (Fig 8A). In addition, a significant negative correlation was observed between the CBF/BOLD ratio and mean baseline CBF for all processing schemes except for HD, PW/E2 which trended toward significance (p = 0.08) (Fig 8B). For the SS technique, CBF/BOLD coupling significantly increased for the PWDN and MECDN datasets compared to the E2 and PW datasets. For the HD technique, CBF/BOLD significantly increased for the MECDN dataset compared to the E2 data (Fig 9). There was a slight, but non significant increase in coupling for the PWDN data for the HD method. These results are also observed in the group maps.

## Discussion

The purpose of this work was to evaluate our recently developed MBME ASL/BOLD sequence for task-fMRI. To accomplish this, a finger-tapping task fMRI study was performed using this sequence. Four total echoes were collected. Combining the echoes resulted in increased BOLD sensitivity. The BOLD data was further cleaned using the MEICA denoising procedure. The MEICA technique was also modified to denoise the PW data. Motor activation was detected from the finger-tapping task using non-denoised and denoised PW and BOLD time-series. Data denoising resulted in increased sensitivity, tSNR, activation strength, and activation volume for the PW and BOLD datasets, and increased CBF/BOLD coupling.

One major advantage of our MBME ASL/BOLD implementation, compared with other typical simultaneous ASL/BOLD sequences, is that it gives us the ability to collect more than two echoes. A T2* weighted combination of the echoes resulted in increased tSNR for the BOLD echoes. MEICA could also be used to denoise the data [23, 24, 27]. The automated MEICA denoising process relies on the TE dependence of BOLD-related ICA components to remove non-BOLD signals from the data. Using MEICA, the tSNR increased approximately 2.5 times compared to E2, resulting in more activation and increased mean t-statistics. Previous work showed increased BOLD sensitivity for resting state fMRI using the MBME ASL/BOLD sequence by combining echoes and incorporating MEICA denoising [12, 30]. Here, we confirm these increases in BOLD sensitivity extend to task-based fMRI as well.

The MECDN data showed increased activation, both in terms of the activation volume and activation strength, compared with the MEC and E2 data, as well as a higher overall tSNR. Though the temporal resolution of the MBME ASL/BOLD sequence was relatively low (TR = 4.0s), the increased tSNR from the additional echoes outweighed the drawbacks of the slight TR increase caused by these echoes. Murphy et al. showed that with increased tSNR, much shorter time-series are needed to detect activations for a certain effect size [44]. In line with this notion, our results also suggest the scan duration could be much shorter by using MECDN or PWDN_art+BOLD_ for fMRI to detect activations with similar effect size as conventional E2 or PW scans. The MBME ASL/BOLD sequence and subsequent denoising strategies provide a trade-off between scan time and activation strength. This is an important implication for future clinical applications.

Moreover, an increased area of activation was present in MECDN data in the group analysis. This included activation in subcortical areas not seen with the MEC and E2 data. Extensive bilateral activation was found in the insula and thalamus. The insula and thalamus have been shown to be part of the somatomotor network [45, 46]. The thalamus is also known to be part of somatosensory pathways, which control our perception of touch. Finger-tapping necessarily involves touch, so activation in these deep gray matter regions should occur. Gradient echo EPI has typically shown poor performance in subcortical regions and requires higher sensitivity to detect significant signal changes [47]. Our results indicate MEICA denoising may be useful for identifying task-based subcortical activation due to the increased tSNR and resulting BOLD sensitivity. Kundu et al. found robust resting-state functional connectivity between subcortical and cortical structures following MEICA denoising, while no clear connectivity patterns could be detected by using standard denoising [24].

In this study, the MEICA algorithm was modified to denoise the PW data by regressing artifact *and* BOLD components from the first echo. BOLD signal can contaminate the PW signal, however, the BOLD and CBF time courses are strongly temporally coupled. Thus, there is the potential for true PW signals to be removed by removing BOLD components. To avoid this, all echoes were low-pass filtered below the label/control oscillation frequency and perfusion frequencies. This removed the majority of the perfusion contribution to the signal while keeping the BOLD contributions. BOLD and artifact components were extracted using this filtered signal and then removed from the unfiltered first echo. To verify PW signal was not being removed, mean PW signal was analyzed. No differences were seen between the denoised and non-denoised data.

It is important to note this denoising technique is limited to block design fMRI studies where the PW signal is relegated to high frequencies, and thus a wideband lowpass filter can be used to remove the PW components. For event-related and resting state studies, this type of filter could potentially include PW components, which will then be removed from the data by the MEICA process.

In this study, both SS and HD techniques were used to extract the PW time-series. While these are two common approaches, they are fundamentally the same. Surround subtraction has been shown to be equivalent to demodulation followed by lowpass filtering, which itself is equivalent to highpass filtering followed by demodulation. We showed PW data quality benefits from the regression of BOLD components in addition to artifact components for both the SS and HD time series. PWDN_ss,art+BOLD_ and PWDN_hd,art+BOLD_ data had increased tSNR and mean t-score compared to PWDN_ss,art_ and PWDN_hd,art_ data respectively (the HD t-score trended toward significance). Both of these techniques have been shown to minimize the contribution of BOLD signal to the ASL signal [4, 38, 42]. These results indicate there may still be BOLD contamination of the PW signal using these methods.

High-pass filtering followed by demodulation was used as a stricter filtering approach to examine if the MEICA denoising technique was still effective. To provide a fair comparison between denoised and non-denoised HD data, the highpass filter frequency was the same as the lowpass filter frequency used for the MEICA denoising approach. By removing low-frequency BOLD and artifact components from the signal, MEICA denoising acts as an imperfect high-pass filter. Thus, in theory, highpass filtering and demodulating the data without denoising should provide similar, if not better, results compared to MEICA denoising as all frequency components below 0.09 Hz, including the inderminate components left by the denoising procedure, are removed. In fact, the HD method without denoising resulted in increased activation strength and volume compared to the SS method with denoising. However, highpass filtering and demodulating the denoised signal did lead to slight, but significant increases in mean t-score and tSNR at the individual level compared to highpass filtering and demodulating the non-denoised signal. SS filtering is not as strict as HD filtering (i.e. the frequency cutoff is lower). Therefore, MEICA denoising has a larger effect as less noise is inherently removed by the SS process, and there is increased BOLD contamination in the signal. Future studies could comprehensively examine the effect of filter cutoff frequency on PW data.

Finally, the relationship between CBF and BOLD activation was investigated. CBF/BOLD response ratios and CBF/BOLD coupling were compared between the denoised data (MECDN and PWDN) and non-denoised data (E2 and PW). The ratio between CBF and BOLD activation magnitude did not change with denoising indicating denoising does not change the quantitative relationship between CBF and BOLD. This has implications for techniques such as BOLD calibration, which uses ASL and BOLD data to estimate CMRO2 [48]. We also found a significantly negative relationship between the CBF/BOLD activation magnitude ratio and baseline CBF that did not change with denoising. This was likely driven by the increased baseline CBF leading to a reduced percent signal change for CBF activation as a larger CBF increase was needed to produce the same percent signal change as a lower baseline CBF. For the SS data, both BOLD and PW denoising with MEICA contributed to increased CBF/BOLD coupling. For the HD data, increases in CBF/BOLD coupling were driven by BOLD denoising with a limited effect from PW denoising.

This study was not without limitations. First, the number of subjects was relatively small. The purpose of this study was to determine the feasibility of using the MBME ASL/BOLD sequence to detect brain activation. Thus, the small subject size was justified. Additionally, only one scan was collected per subject, so a repeatability analysis could not be conducted. Future studies should look at the repeatability/reproducibility of MBME ASL/BOLD scans.

A relatively short PLD (1.5s) was employed for this study compared to the recommended PLD of 1.8 s for pCASL [11]. Shorter PLDs have the benefit of increased SNR, but can lead to intravascular artifacts if blood does not have adequate time to reach capillary-feeding small arteries. In addition, when the PLD is too short, the CBF response to activation can be overestimated. For example, if the arterial transit time is reduced in the activated state, the proportion of tagged blood in the voxel will be higher creating an artificially increased change in CBF [49]. This could impact studies where quantitative accuracy of CBF is necessary and should be examined in future studies. The PLD chosen here was a trade-off between TR, SNR, and the arterial transit time.

The issue of short PLD is especially important for interleaved MB acquisitions with MB slice packets that simultaneously cover inferior and superior slices. Intravascular artifacts were not detected in this study, and we were also able to consistently detect PW activation. Looking into locations for the simultaneously excited slices in MB pCASL may be worthwhile. Increasing PLD necessarily increases TR. The TR for ASL acquisitions is already long, owing to the long tagging and PLD segments of the sequence. However, we have shown that additional echoes collected in the MBME ASL/BOLD sequence can compensate for the longer TR.

Of note, most recently developed ASL sequences have utilized a segmented 3D readout, such as 3D GRASE, or stack of spirals approaches [11]. These techniques can also acquire whole-brain, high resolution ASL data; however, they are not compatible with multi-echo (ME) time-series acquisitions as they lead to very long TEs (for the 3D GRASE case) or TRs (for the segmented stack of spirals case). Background suppression (BS) was not used for the ASL scans in this study. There remains debate whether BS is appropriate for 2D approaches since BS can only be optimized for one slice. Furthermore, BS necessarily reduces BOLD SNR and tSNR. Future studies should examine the effect of BS on MBME ASL/BOLD.

In conclusion, we applied the MBME ASL/BOLD sequence to task fMRI. Motor activation was robustly detected using ASL and BOLD. In addition, the collection of more than two echoes allowed MEICA denoising to be applied to both the BOLD and PW data, resulting in increased tSNR, activation strength and volume, and CBF/BOLD coupling.

## Abbreviations

ASL: Arterial spin labeling
BCP: BOLD constrained perfusion
BOLD: Blood oxygenation-level dependent
BS: Background suppression
CBF: Cerebral blood flow
CBV: Cerebral blood volume
CMRO2: Cerebral metabolic rate of oxygen consumption
CVR: Cerebrovascular reactivity
EPI: Echo planar imaging
FWHM: Full width at half maximum
GLM: General linear model
GRAPPA: Generalized autocalibrating partially parallel acquisitions
ICA: Independent component analysis
MB: Multiband
MBME: Multiband multi-echo
ME: Multi-echo
MEC: Multi-echo combined
MEICA: ME independent component analysis
MECDN: MEICA denoised multi-echo
MNI: Montreal Neurological Institute
MPRAGE: Magnetization-prepared rapid acquisition with gradient echo
pCASL: Pseudo-continuous
PLD: Post-labeling delay
PW: Perfusion-weighted
PWDN: Perfusion-weighted denoised
SB: Single band
SE: Single echo
SNR: Signal-to-noise ratio
tSNR: Temporal signal-to-noise ratio

## References

[1] Ghariq E, Chappell MA, Schmid S, Teeuwisse WM, van Osch MJP. Effects of background suppression on the sensitivity of dual-echo arterial spin labeling MRI for BOLD and CBF signal changes. NeuroImage. 2014;103:316–22. 10.1016/j.neuroimage.2014.09.051. 25280450

[2] Schmithorst VJ, Hernandez-Garcia L, Vannest J, Rajagopal A, Lee G, Holland SK. Optimized simultaneous ASL and BOLD functional imaging of the whole brain. Journal of magnetic resonance imaging: JMRI. 2014;39(5):1104–17. Epub 2013/10/12. 10.1002/jmri.24273 24115454 PMC3964152

[3] Tak S, Polimeni JR, Wang DJ, Yan L, Chen JJ. Associations of Resting-State fMRI Functional Connectivity with Flow-BOLD Coupling and Regional Vasculature. Brain connectivity. 2015;5(3):137–46. 10.1089/brain.2014.0299 25384681 PMC4394176

[4] Tak S, Wang DJJ, Polimeni JR, Yan L, Chen JJ. Dynamic and static contributions of the cerebrovasculature to the resting-state BOLD signal. NeuroImage. 2014;84:672–80. 10.1016/j.neuroimage.2013.09.057. 24099842 PMC4323159

[5] Zhu S, Fang Z, Hu S, Wang Z, Rao H. Resting state brain function analysis using concurrent BOLD in ASL perfusion fMRI. PloS one. 2013;8(6):e65884 10.1371/journal.pone.0065884 23750275 PMC3672100

[6] Whittaker JR, Driver ID, Bright MG, Murphy K. The absolute CBF response to activation is preserved during elevated perfusion: Implications for neurovascular coupling measures. NeuroImage. 2016;125:198–207. Epub 2015/10/21. 10.1016/j.neuroimage.2015.10.023 26477657 PMC4692513

[7] Bangen KJ, Restom K, Liu TT, Jak AJ, Wierenga CE, Salmon DP, et al Differential age effects on cerebral blood flow and BOLD response to encoding: associations with cognition and stroke risk. Neurobiol Aging. 2009;30(8):1276–87. 10.1016/j.neurobiolaging.2007.11.012 18160181 PMC2804245

[8] Restom K, Bangen KJ, Bondi MW, Perthen JE, Liu TT. Cerebral blood flow and BOLD responses to a memory encoding task: a comparison between healthy young and elderly adults. NeuroImage. 2007;37(2):430–9. Epub 2007/06/26. 10.1016/j.neuroimage.2007.05.024 17590353 PMC2214854

[9] Faraco CC, Strother MK, Dethrage LM, Jordan L, Singer R, Clemmons PF, et al Dual echo vessel-encoded ASL for simultaneous BOLD and CBF reactivity assessment in patients with ischemic cerebrovascular disease. Magnetic resonance in medicine: official journal of the Society of Magnetic Resonance in Medicine / Society of Magnetic Resonance in Medicine. 2015;73(4):1579–92. Epub 2014/04/24. 10.1002/mrm.25268 . 24757044 PMC4435663

[10] De Vis JB, Hendrikse J, Bhogal A, Adams A, Kappelle LJ, Petersen ET. Age-related changes in brain hemodynamics; A calibrated MRI study. Hum Brain Mapp. 2015;36(10):3973–87. 10.1002/hbm.22891 . 26177724 PMC6869092

[11] Alsop DC, Detre JA, Golay X, Gunther M, Hendrikse J, Hernandez-Garcia L, et al Recommended implementation of arterial spin-labeled perfusion MRI for clinical applications: A consensus of the ISMRM perfusion study group and the European consortium for ASL in dementia. Magn Reson Med. 2015;73(1):102–16. 10.1002/mrm.25197 24715426 PMC4190138

[12] Cohen AD, Nencka AS, Lebel RM, Wang Y. Multiband multi-echo imaging of simultaneous oxygenation and flow time-series for resting state connectivity. PloS one. 2017;12(3):e0169253 10.1371/journal.pone.0169253 28253268 PMC5333818

[13] Chen L, TV A, Xu J, Moeller S, Ugurbil K, Yacoub E, et al Evaluation of highly accelerated simultaneous multi-slice EPI for fMRI. Neuroimage. 2015;104:452–9. 10.1016/j.neuroimage.2014.10.027 25462696 PMC4467797

[14] Feinberg DA, Setsompop K. Ultra-fast MRI of the human brain with simultaneous multi-slice imaging. J Magn Reson. 2013;229:90–100. 10.1016/j.jmr.2013.02.002 23473893 PMC3793016

[15] Moeller S, Yacoub E, Olman CA, Auerbach E, Strupp J, Harel N, et al Multiband multislice GE-EPI at 7 tesla, with 16-fold acceleration using partial parallel imaging with application to high spatial and temporal whole-brain fMRI. Magn Reson Med. 2010;63(5):1144–53. 10.1002/mrm.22361 20432285 PMC2906244

[16] Setsompop K, Gagoski BA, Polimeni JR, Witzel T, Wedeen VJ, Wald LL. Blipped-controlled aliasing in parallel imaging for simultaneous multislice echo planar imaging with reduced g-factor penalty. Magn Reson Med. 2012;67(5):1210–24. 10.1002/mrm.23097 21858868 PMC3323676

[17] Feinberg DA, Beckett A, Chen L. Arterial spin labeling with simultaneous multi-slice echo planar imaging. Magnetic resonance in medicine: official journal of the Society of Magnetic Resonance in Medicine / Society of Magnetic Resonance in Medicine. 2013;70(6):1500–6. Epub 2013/10/17. 10.1002/mrm.24994 24130105 PMC4162886

[18] Kim T, Shin W, Zhao T, Beall EB, Lowe MJ, Bae KT. Whole brain perfusion measurements using arterial spin labeling with multiband acquisition. Magn Reson Med. 2013;70(6):1653–61. Epub 2013/07/24. 10.1002/mrm.24880 . 23878098

[19] Wang Y, Moeller S, Li X, Vu AT, Krasileva K, Ugurbil K, et al Simultaneous multi-slice Turbo-FLASH imaging with CAIPIRINHA for whole brain distortion-free pseudo-continuous arterial spin labeling at 3 and 7 T. NeuroImage. 2015;113:279–88. Epub 2015/04/04. 10.1016/j.neuroimage.2015.03.060 25837601 PMC4433786

[20] Poser BA, Norris DG. Investigating the benefits of multi-echo EPI for fMRI at 7 T. NeuroImage. 2009;45(4):1162–72. 10.1016/j.neuroimage.2009.01.007. 19349231

[21] Posse S. Multi-echo acquisition. NeuroImage. 2012;62(2):665–71. 10.1016/j.neuroimage.2011.10.057. 22056458 PMC3309060

[22] Kundu P, Benson B, Baldwin K, Rosen D, Luh W-M, Bandettini P, et al Robust resting state fMRI processing for studies on typical brain development based on multi-echo EPI acquisition. Brain Imaging and Behavior. 2015;9(1):56–73. 10.1007/s11682-014-9346-4 25592183 PMC6319659

[23] Kundu P, Brenowitz ND, Voon V, Worbe Y, Vertes PE, Inati SJ, et al Integrated strategy for improving functional connectivity mapping using multiecho fMRI. Proc Natl Acad Sci U S A. 2013;110(40):16187–92. 10.1073/pnas.1301725110 24038744 PMC3791700

[24] Kundu P, Inati SJ, Evans JW, Luh WM, Bandettini PA. Differentiating BOLD and non-BOLD signals in fMRI time series using multi-echo EPI. Neuroimage. 2012;60(3):1759–70. 10.1016/j.neuroimage.2011.12.028 22209809 PMC3350785

[25] Kundu P, Santin MD, Bandettini PA, Bullmore ET, Petiet A. Differentiating BOLD and non-BOLD signals in fMRI time series from anesthetized rats using multi-echo EPI at 11.7 T. Neuroimage. 2014;102 Pt 2:861–74. 10.1016/j.neuroimage.2014.07.025 . 25064668

[26] Poser BA, Versluis MJ, Hoogduin JM, Norris DG. BOLD contrast sensitivity enhancement and artifact reduction with multiecho EPI: parallel-acquired inhomogeneity-desensitized fMRI. Magn Reson Med. 2006;55(6):1227–35. 10.1002/mrm.20900 . 16680688

[27] Evans JW, Kundu P, Horovitz SG, Bandettini PA. Separating slow BOLD from non-BOLD baseline drifts using multi-echo fMRI. NeuroImage. 2015;105:189–97. 10.1016/j.neuroimage.2014.10.051. 25449746 PMC4262662

[28] Dai W, Garcia D, de Bazelaire C, Alsop DC. Continuous flow-driven inversion for arterial spin labeling using pulsed radio frequency and gradient fields. Magn Reson Med. 2008;60(6):1488–97. 10.1002/mrm.21790 19025913 PMC2750002

[29] Wu WC, Fernandez-Seara M, Detre JA, Wehrli FW, Wang J. A theoretical and experimental investigation of the tagging efficiency of pseudocontinuous arterial spin labeling. Magn Reson Med. 2007;58(5):1020–7. 10.1002/mrm.21403 . 17969096

[30] Suchandrima Banerjee AT, Yuval Zur, Ajit Shankaranarayanan, Douglas A.C. Kelley, editor Robust calibration strategy for multiband EPI at 7 Tesla. International Society for Magnetic Resonance in Medicine; 2012; Melbourne, Australia.

[31] Griswold MA, Jakob PM, Heidemann RM, Nittka M, Jellus V, Wang J, et al Generalized autocalibrating partially parallel acquisitions (GRAPPA). Magn Reson Med. 2002;47(6):1202–10. 10.1002/mrm.10171 . 12111967

[32] Noll DC, Nishimura DG, Macovski A. Homodyne detection in magnetic resonance imaging. IEEE Trans Med Imaging. 1991;10(2):154–63. 10.1109/42.79473 . 18222812

[33] Cox RW. AFNI: software for analysis and visualization of functional magnetic resonance neuroimages. Comput Biomed Res. 1996;29(3):162–73. Epub 1996/06/01. . 8812068 10.1006/cbmr.1996.0014

[34] Cox RW, Hyde JS. Software tools for analysis and visualization of fMRI data. NMR Biomed. 1997;10(4–5):171–8. . 9430344 10.1002/(sici)1099-1492(199706/08)10:4/5<171::aid-nbm453>3.0.co;2-l

[35] Jenkinson M, Beckmann CF, Behrens TE, Woolrich MW, Smith SM. Fsl. Neuroimage. 2012;62(2):782–90. 10.1016/j.neuroimage.2011.09.015 . 21979382

[36] Jenkinson M, Bannister P, Brady M, Smith S. Improved optimization for the robust and accurate linear registration and motion correction of brain images. Neuroimage. 2002;17(2):825–41. . 12377157 10.1016/s1053-8119(02)91132-8

[37] Posse S, Wiese S, Gembris D, Mathiak K, Kessler C, Grosse-Ruyken ML, et al Enhancement of BOLD-contrast sensitivity by single-shot multi-echo functional MR imaging. Magn Reson Med. 1999;42(1):87–97. . 10398954 10.1002/(sici)1522-2594(199907)42:1<87::aid-mrm13>3.0.co;2-o

[38] Liu TT, Wong EC. A signal processing model for arterial spin labeling functional MRI. NeuroImage. 2005;24(1):207–15. Epub 2004/12/14. 10.1016/j.neuroimage.2004.09.047 . 15588612

[39] Lu H, Donahue MJ, van Zijl PC. Detrimental effects of BOLD signal in arterial spin labeling fMRI at high field strength. Magn Reson Med. 2006;56(3):546–52. 10.1002/mrm.20976 . 16894581

[40] Aguirre GK, Detre JA, Zarahn E, Alsop DC. Experimental design and the relative sensitivity of BOLD and perfusion fMRI. NeuroImage. 2002;15(3):488–500. Epub 2002/02/19. 10.1006/nimg.2001.0990 . 11848692

[41] Wong EC, Buxton RB, Frank LR. Implementation of quantitative perfusion imaging techniques for functional brain mapping using pulsed arterial spin labeling. NMR in biomedicine. 1997;10(4–5):237–49. . 9430354 10.1002/(sici)1099-1492(199706/08)10:4/5<237::aid-nbm475>3.0.co;2-x

[42] Chuang KH, van Gelderen P, Merkle H, Bodurka J, Ikonomidou VN, Koretsky AP, et al Mapping resting-state functional connectivity using perfusion MRI. NeuroImage. 2008;40(4):1595–605. Epub 2008/03/04. 10.1016/j.neuroimage.2008.01.006 18314354 PMC2435272

[43] Cox RW, Chen G, Glen DR, Reynolds RC, Taylor PA. FMRI Clustering in AFNI: False-Positive Rates Redux. Brain connectivity. 2017;7(3):152–71. Epub 2017/04/12. 10.1089/brain.2016.0475 28398812 PMC5399747

[44] Murphy K, Bodurka J, Bandettini PA. How long to scan? The relationship between fMRI temporal signal to noise ratio and necessary scan duration. Neuroimage. 2007;34(2):565–74. 10.1016/j.neuroimage.2006.09.032 17126038 PMC2223273

[45] Lee MH, Hacker CD, Snyder AZ, Corbetta M, Zhang D, Leuthardt EC, et al Clustering of resting state networks. PloS one. 2012;7(7):e40370 10.1371/journal.pone.0040370 22792291 PMC3392237

[46] Carter AR, Astafiev SV, Lang CE, Connor LT, Rengachary J, Strube MJ, et al Resting interhemispheric functional magnetic resonance imaging connectivity predicts performance after stroke. Ann Neurol. 2010;67(3):365–75. 10.1002/ana.21905 20373348 PMC2927671

[47] Lowe MJ, Lurito JT, Mathews VP, Phillips MD, Hutchins GD. Quantitative comparison of functional contrast from BOLD-weighted spin-echo and gradient-echo echoplanar imaging at 1.5 Tesla and H2 15O PET in the whole brain. J Cereb Blood Flow Metab. 2000;20(9):1331–40. 10.1097/00004647-200009000-00008 . 10994855

[48] Davis TL, Kwong KK, Weisskoff RM, Rosen BR. Calibrated functional MRI: mapping the dynamics of oxidative metabolism. Proceedings of the National Academy of Sciences of the United States of America. 1998;95(4):1834–9. 9465103 10.1073/pnas.95.4.1834 PMC19199

[49] Gonzalez-At JB, Alsop DC, Detre JA. Cerebral perfusion and arterial transit time changes during task activation determined with continuous arterial spin labeling. Magnetic resonance in medicine: official journal of the Society of Magnetic Resonance in Medicine / Society of Magnetic Resonance in Medicine. 2000;43(5):739–46. Epub 2000/05/09. . 10800040 10.1002/(sici)1522-2594(200005)43:5<739::aid-mrm17>3.0.co;2-2

